# Supplementary material for: Awareness, attitudes, and beliefs about palliative care: Results from a representative survey of the Italian-speaking Swiss population
Source: PLoS One. 2023 Nov 28;18(11):e0294807. doi: 10.1371/journal.pone.0294807 (PMC10684106; doi:10.1371/journal.pone.0294807)
Supplement: Attachment — Submitted filename: Response to reviewers.docx [file pone.0294807.s001.docx]

Lucerne, November 9^th^, 2023

Dear Dr. Brunelli, Dear Reviewers,

We would like to express our heartfelt gratitude for your time and effort in reviewing our manuscript titled **Awareness, Attitudes, and Beliefs about Palliative Care: Results from a Representative Survey of the Italian-Speaking Swiss Population**. Your comments have been extremely constructive, and we have addressed each point in detail below. We have revised our manuscript accordingly to enhance its clarity and robustness.

In addition, we have added the requested information in order to adhere to the journal’s requirements. Specifically:

1. We edited the manuscript to ensure it meets PLOS ONE's style requirements.
2. We amended our current ethics statement to show that written informed consent was provided.
3. We checked the reference list to ensure that it is complete and correct.

We confirm once again that this manuscript has not been published elsewhere and is not under consideration by another journal.

All authors have approved the revised manuscript and agree with its resubmission to PLOS ONE.

We look forward to hearing from you at your earliest convenience.

Best Regards,

Nicola Diviani, PhD (corresponding author)

[Nicola.Diviani@paraplegie.ch](mailto:Nicola.Diviani@paraplegie.ch)

**Point-by-point response to reviewers’ comments**

| **Reviewer 1** |  |
| --- | --- |
| Thank you for giving me the opportunity to review this well-written and relevant article - the results are very interesting and clearly described. I have a few comments: | We thank Reviewer 1 for the appreciation of our work. |
| the authors use "PC" as abbreviation without prior explanation - please add or revise | Thank you for pointing this out. We decided to avoid using the abbreviation altogether. |
| some sentences are rather long or complicated (e.g. chapter 1 Introduction 3rd paragraph starting with "in addition...", chapter 4.1 4th paragraph starting with "the third set of considerations... 2nd sentence is rather long, 5th paragraph starting with "the previous points..." 2nd sentence why "instead"? - please revise | We thank the reviewer for pointing out that some sentences were rather long or complicated. We took care to revise them and to split them in several shorter sentences where needed. |
| ad 2.1 sample: please give some more information on how this "internet panel" works and please dicscuss the limitation of such a population because especially the elderly population might not have access to this panel which might limit the interpretation | Thank you for this suggestion. We revised the Methods section to add more information on the panel. We now also acknowledge the possible limitation linked to the choice of such a population at the end of the discussion section. |
| ad 2.2.1. - if respondents never heard of the term - where they redirected to the socio-demographic section or the health-related information-seeking section? why where they not asked to fill in section iv? also | Thank you for this question. They were indeed redirected to the health-related information-seeking questions (section iv). We realized that we incorrectly described it as the last section of the questionnaire, and this might have caused confusion. We have now revised the sentence. |
| - could the authors state why they chose the "facts and (false) beliefs which they mentioned - there might be others too and the authors made a selection | Thank you for this question. The statements were chosen based on established definitions of palliative care, e.g., by the World Health Organization, and on common myths that have been described in the literature. We now mention this aspect in the Methods section. |
| ad 2.2.5. - why did you ask about "religiosity" - this was not mentioned as potentially relevant in your introduction? Religiosity was also not analysed as relevant factor (compared to gender, ...) - was it in any way relevant? | Thank you for pointing this out. We included “religiosity” as an exploratory variable based on prior literature suggesting a possible link with attitudes toward palliative care. However, it was not found to be significant in our analyses and hence was not discussed in detail. |
| ad 2.3 - based on which methodological approach did the authors perform the thematic analysis? | Thank you for this question. We used the methodological approach described by Braun & Clarke (2006). We added a few details and a reference in the Data analysis section. |
| - ad 4.1 discussion: could you explain more, what a "dedicated information point" is? | Thank you for this question. A "dedicated information point" for palliative care is a centralized resource, either in a physical location or accessible online, where individuals can obtain comprehensive non-medical information related to palliative care. This resource aims to educate and support patients, families, and caregivers by providing details on palliative services, guidance on navigating care options, legal and ethical considerations, psychological support networks, and practical advice for day-to-day care. We have added some more details in the Discussion section to improve clarity. |
| could the authors add the references to the last sentence in the discussion in which they refer to "...similar studies on the topic..." | Thank you for this suggestion. We added a few exemplar references. |
| - Table 3: I would find it more helpful if the authors could sort the answers to questions 2-4 based on the frequency. Just out of curiosity: what are the thoughts of the authors that most respondents did not wish any additional information about palliative care? | Thank you for this suggestion. We have reorganized the answers to questions 2-4 based on frequency, with sources and channels more frequently mentioned listed first.  As for your question regarding possible reasons for not wanting additional information about palliative care, we thought about two main reasons. First, respondents may believe they have enough knowledge or may not see the immediate relevance of palliative care to their lives, leading to a perceived lack of need for further information. Alternatively, or additionally, the association of palliative care with end-of-life may create discomfort or avoidance, as individuals are often reluctant to engage with topics that remind them of mortality or serious illness. |
| - could the authors add specific details to the references e.g. as to reference 1 (access specifics are needed) | Thank you for this suggestion. We checked the reference list and added specific details where needed. Reference 1 is a book and |
| **Reviewer 2** |  |
| I don't have other comments. | We thank Reviewer 2 for the positive evaluation of our manuscript. |
